# Genome-Wide Scan for Visceral Leishmaniasis in Mixed-Breed Dogs Identifies Candidate Genes Involved in T Helper Cells and Macrophage Signaling

**DOI:** 10.1371/journal.pone.0136749

**Published:** 2015-09-08

**Authors:** Yuri T. Utsunomiya, Érica S. Ribeiro, Amanda P. N. Quintal, Juliano R. Sangalli, Valquiria R. Gazola, Henrique B. Paula, Cristiana M. Trinconi, Valéria M. F. Lima, Silvia H. V. Perri, Jeremy F. Taylor, Robert D. Schnabel, Tad S. Sonstegard, José F. Garcia, Cáris M. Nunes

**Affiliations:** 1 Departamento de Medicina Veterinária Preventiva e Reprodução Animal, Faculdade de Ciências Agrárias e Veterinárias, UNESP—Univ Estadual Paulista, Jaboticabal, São Paulo, Brazil; 2 Departamento de Apoio, Saúde e Produção Animal, Faculdade de Medicina Veterinária de Araçatuba, UNESP—Univ Estadual Paulista, Araçatuba, São Paulo, Brazil; 3 Division of Animal Sciences, University of Missouri, Columbia, 65211, United States of America; 4 Animal Genomics and Improvement Laboratory, ARS-USDA—Agricultural Research Service—United States Department of Agriculture, Beltsville, Maryland, United States of America; University of São Paulo, BRAZIL

## Abstract

We conducted a genome-wide scan for visceral leishmaniasis in mixed-breed dogs from a highly endemic area in Brazil using 149,648 single nucleotide polymorphism (SNP) markers genotyped in 20 cases and 28 controls. Using a mixed model approach, we found two candidate loci on canine autosomes 1 and 2. The positional association on chromosome 2 mapped to a predicted DNAse sensitive site in CD14+ monocytes that serve as a cis-regulatory element for the expression of interleukin alpha receptors 2 (*IL2RA*) and 15 (*IL15RA*). Both interleukins were previously found to lead to protective T helper 1 cell (Th1) response against *Leishmania spp*. in humans and mice. The associated marker on chromosome 1 was located between two predicted transcription factor binding sites regulating the expression of the transducin-like enhancer of split 1 gene (*TLE1*), an important player in Notch signaling. This pathway is critical for macrophage activity and CD4+ T cell differentiation into Th1 and T helper 2. Together, these findings suggest that the human and mouse model for protective response against *Leishmania spp*., which involves Th1 and macrophage modulation by interleukins 2, 15, gamma interferon and Notch signaling, may also hold for the canine model.

## Introduction

The domestic dog (*Canis lupus familiaris*) is the primary urban reservoir of visceral leishmaniasis (VL), a neglected tropical disease that affects about 500,000 people every year [1]. Susceptibility to VL has been reported in several dog breeds, such as the Boxer, Doberman, German Shepherd and Cocker Spaniel, indicating the existence of an important, yet not understood, genetic component underlying host-parasite interplay [2–4].

Quilez et al. (2012) [5] estimated that additive genetic variance could account for as much as 64% of the total variance in VL clinical manifestations in a sample of Boxer dogs, and showed that the clinical outcome of 60% of the analyzed infected dogs could correctly be predicted using a panel of 126,607 genome-wide single nucleotide polymorphism (SNP) markers. Although candidate genes implicated in VL have been proposed in both humans and dogs [4,6,7], evidence from agnostic genome-wide scans remains limited to only a few reports [5,8].

Here, we used over 145,000 SNPs distributed throughout the canine genome to identify genes involved in *Leishmania spp*. infection in random mating mixed-breed (mongrel) dogs originated from an area highly endemic for VL (Araçatuba, São Paulo–Brazil).

## Results and Discussion

**Fig 1:**
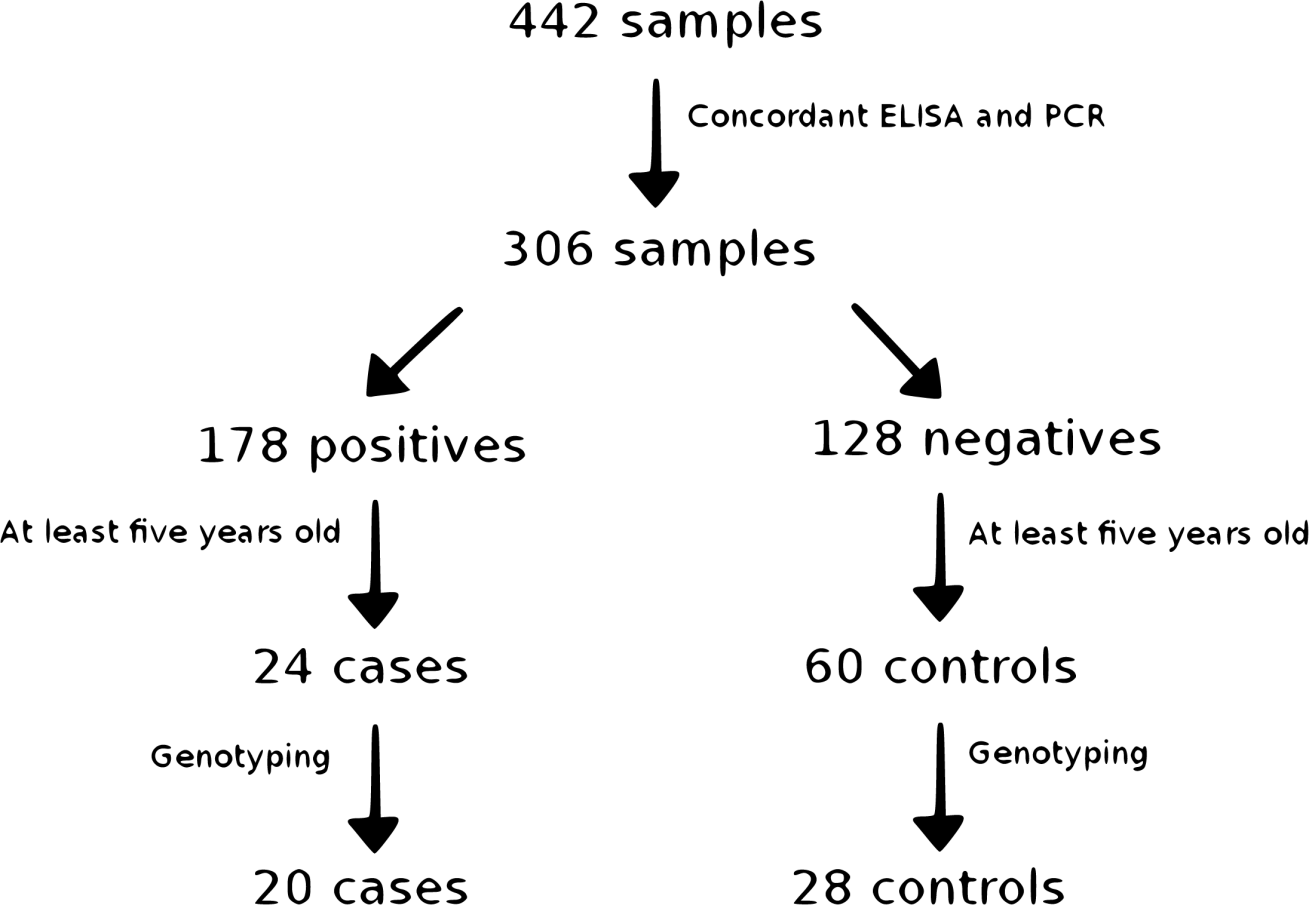
Sample screening for *Leishmania spp*. infection in mixed-breed dogs. Samples were selected to maximize the likelihood of exposure (by age) and correct infection diagnosis (by concordance of diagnostic tests). Balanced numbers of cases and controls were genotyped and quality-controlled.

**Fig 1** summarizes the sample screening process conducted in this study. Of the 442 sampled animals, 223 (50.5%) tested positive for *Leishmania spp*. by Polymerase Chain Reaction (PCR) and 168 (38.4%) by Indirect Enzyme-linked Immunosorbent Assay (ELISA). Considering positive status in at least one of the diagnostic tests, we observed 263 positive dogs (59.5%).

To evaluate genotype-phenotype associations, we selected samples for which the PCR and ELISA results were concordant, which reduced the number of samples to 306, with 178 (58.2%) testing negative and 128 (41.8%) testing positive. Additionally, in order to maximize the likelihood of exposure in the sample, we considered cases and controls only the animals that presented at least five years of age. Therefore, a total of 24 cases and 60 controls were identified. In order to balance the number of cases and controls, we randomly genotyped 20 cases and 28 controls with the Illumina CanineHD BeadChip.

**Fig 2:**
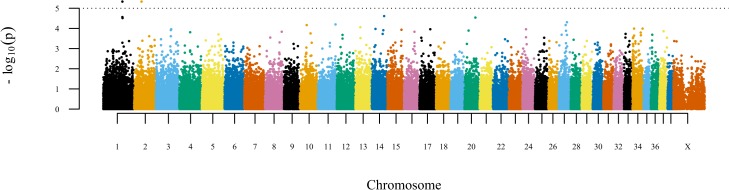
A genome-wide scan using a variance components model in mixed-breed dogs identifies two loci associated with infection by *Leishmania spp*. (p < 1 x 10^−5^). The markers on autosomes 1 (rs22039047, p = 4.6 x 10^−6^) and 2 (rs22840096, p = 4.7 x 10^−6^) presented estimated B allele (Illumina A/B genotype calls) odds ratios of 16.12 ± 2.09 and 37.34 ± 3.09, respectively.

From the initial set of 173,661 markers, 149,648 (86.2%) passed all inclusion criteria (see Material and Methods), and no individual was excluded due to low call rate (<90%). We performed a genome-wide scan in dogs with positive and negative diagnostic tests for *Leishmania spp*. infection using a mixed model approach [9]. The genomic inflation factor was approximately 1, indicating that confounding due to population structure and cryptic relatedness were dully controlled in the analysis. Two markers were declared significant (p < 1 x 10^−5^), representing two different loci on autosomes 1 and 2 (**Fig 2**).

**Fig 3:**
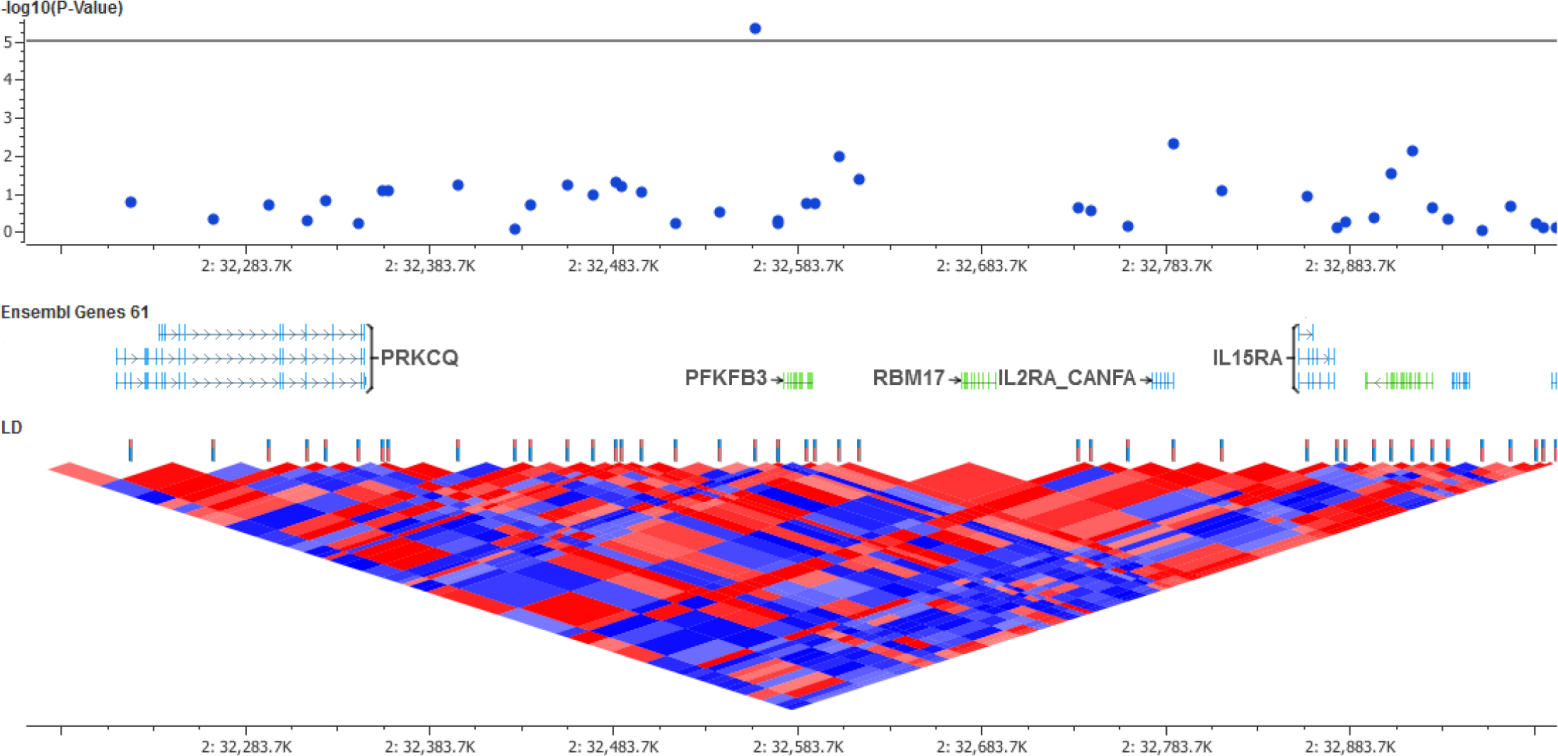
Regional plot of the chromosome 2 marker (rs22840096) associated with *Leishmania spp*. infection in mixed-breed dogs. Of note, the marker is located approximately 200 kb from *IL2RA* and *IL15RA* in a predicted CD14+ monocyte DNAse hypersensitive site. At the bottom, the D' measure of linkage disequilibrium shows the extent of SNP tagging nearby the positional association.

The marker mapping at 32,560,323 bp on chromosome 2 (rs22840096, p = 4.7 x 10^−6^, see **Fig 3**) presented a B allele frequency (Illumina A/B allele coding) of 43% in cases and of 7% in controls, and an estimated odds ratio of 16.12 ± 2.09. This positional association was found approximately 200 kb upstream of *IL2RA* and *IL15RA*, which encode for interleukin 2 (IL-2) and 15 (IL-15) alpha receptors, respectively. Also, the variant overlapped a predicted CD14+ monocyte DNAse hypersensitive site, which suggests that the positional candidate may be capturing the signal of a variant cis-regulating the expression of downstream genes *IL2RA* and *IL15RA* in macrophages. Cytokines IL-2 and gamma interferon, which are mainly produced by T helper 1 (Th1) cells, are related to protection against *Leishmania spp*. [10]. In fact, Th1 and T helper 2 (Th2) responses, presumably mediated by cytokines such as IL-2 and gamma interferon, were respectively found to be associated with resistance and susceptibility against *Leishmania spp*. in the mouse model [11]. Also, IL-15 is mainly produced by monocytes and was previously found to be protective by inducing the production of IL-12 and consequently Th1 celular response, leading to parasite elimination in infected cells [12,13]. The complex immune response against *Leishmania spp*. parasites in dogs is still superficially understood, but the functional candidate genes found in the vicinity of our positional association suggest that these interleukins also play an important role in the control and resolution of the infection in the canine model.

**Fig 4:**
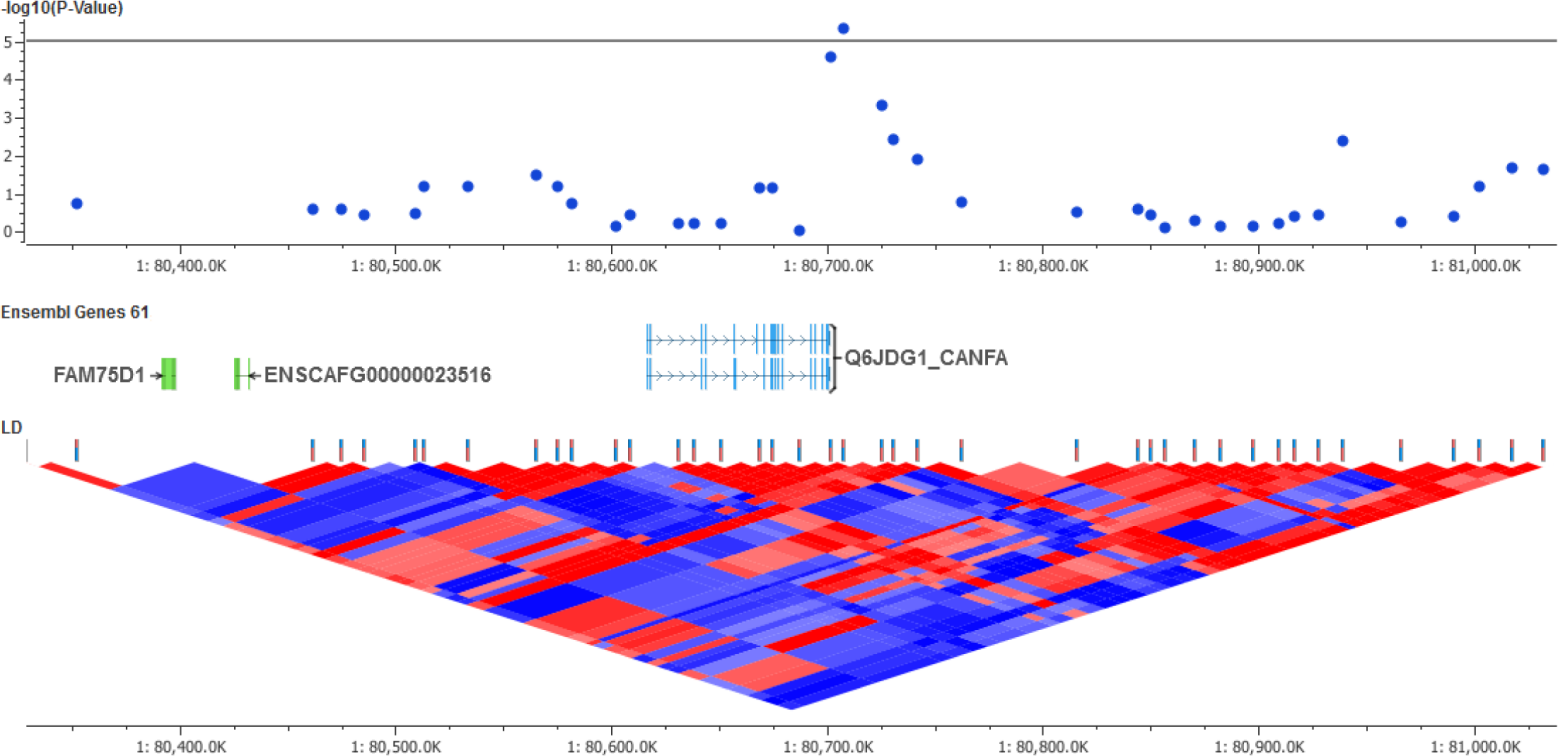
Regional plot of the chromosome 1 marker (rs22039047) associated with *Leishmania spp*. infection in mixed-breed dogs. The marker is in the vicinity of a predicted transcription factor binding site controlling the expression of *TLE1 (Q6JDG1_CANFA)*. At the bottom, the D' measure of linkage disequilibrium shows the extent of SNP tagging nearby the positional association.

The marker on chromosome 1 at position 80,707,336 (rs22039047, p = 4.6 x 10^−6^, see **Fig 4**) exhibited an estimated odds ratio of 37.34 ± 3.09 and a B allele frequency of 32.5% in cases and 1.8% in controls. Comparative genomics analysis using ENCODE and UCSC data suggested that the marker is between two transcription factor binding sites regulating the expression of *TLE1* (transducin-like enhancer of split 1). This gene is also known as *Q6JDG1_CANFA* and participates in the Notch signaling pathway [14], which is critical for macrophage activity and CD4+ T cell differentiation. Mice lacking notch signaling in CD4+ T cells were shown to have defective Th2 cell differentiation, but they were able to differentiate in Th1 cells and control *Leishmania major* infection [15]. Also, Notch receptors were shown to be necessary for gamma interferon production by murine Th1 cells during *Leishmania major* infection [16]. Interestingly, the positional associations on chromosomes 1 and 2 found in the present study point to the same resistance mechanism, which involves parasite killing triggered by T helper 1 cells and macrophage signaling.

## Conclusions

We identified positional candidate loci for *Leishmania spp*. infection in dogs, which shelter functional candidate genes participating in important molecular mechanisms underlying parasite elimination. This preliminary study involved a small sample of mongrel dogs, and the findings reported here require replication to confirm and further elucidate the role of these loci in visceral leishmaniasis. Fine mapping these candidate genes using re-sequencing data may contribute to the identification of variants implicated in susceptibility to visceral leishmaniasis in dogs.

## Materials and Methods

### Sampling and Study Area

The study was conducted in the municipality of Araçatuba (São Paulo state, Brazil), an area highly endemic for canine VL since 1999. Dog blood samples were obtained from two different sources: 1) dogs seen at the Veterinary Hospital of the Faculty of Veterinary Medicine of Araçatuba (UNESP); or 2) dogs submitted for euthanasia at the Center for Zoonosis Control of Araçatuba. This study was carried out in strict accordance with the recommendations in the Ethical Principles of the Brazilian College of Animal Experimentation (COBEA– http://www.cobea.org.br). The protocol was approved by the UNESP Ethics Committee on Animal Experimentation (CEUA-FOA permit number: 2008–001309). Written informed consent was obtained from all participant owners. We sampled a total of 442 dogs over two years of age, assuming that they had been naturally exposed to the parasite based upon the extremely high disease prevalence and minimum age of exposure to *Leishmania spp*. in the study area [17].

### Infection Status

Amplification of *Leishmania spp*. kinetoplast DNA: Purification of DNA from canine blood samples was performed by the phenol/chloroform/isoamyl alcohol method [18]. Detection of parasite DNA was based on the primers described by Rodgers et al. (1990) [19], which amplify a 120-bp conserved region of the *Leishmania spp*. kinetoplast. A positive control (DNA sample from a dog with positive serological diagnosis and clinical signs of leishmaniasis), a negative control (DNA sample from a dog with negative serological diagnosis and no clinical signs) and a negative control reaction (no DNA) were included in each PCR amplification. The PCR products were visualized by gel electrophoresis in 8% polyacrylamide gels stained with silver nitrate solution.

Indirect Enzyme-linked immunosorbent assay (ELISA): The ELISA test was conducted according to Lima et al. (2005) [20]. Positive (case) or negative (control) leishmaniasis status was determined for those dogs with at least 5 years of age that had identical diagnostic results for the PCR and ELISA tests.

### Genome-Wide SNP Genotyping

A balanced subset of the identified cases and controls was randomly chosen and genotyped for 173,661 SNP markers using the Illumina CanineHD BeadChip, according to the manufacturer's protocol.

### Association Analysis

We used the SNP & Variation Suite 8 (SVS–Golden Helix Inc., available at: http://goldenhelix.com/SNP_Variation/) software to test for phenotype-genotype associations via a linear mixed model controlled for random polygenic effects [9]. Prior to the SNP association analysis, genotypic data were filtered based on the following inclusion criteria: SNP call rate greater than 95%, SNP minor allele frequency (MAF) greater than 5%, SNP p-value for Hardy-Weinberg Equilibrium (HWE) exact test greater than 10^−5^, and sample call rate greater than 90%. Markers were prioritized for investigation based on a significance threshold of p < 1 x 10^−5^. Log-odds ratios for these markers were estimated based on the slopes from a generalized linear model assuming a binomial distribution and a logit link function using *glm()* in R v.3.1.0 (available at: http://www.r-project.org/).

### Gene and Regulatory Elements Annotation

Genomic regions harboring significant signals were visually inspected for gene annotation using the GoldenHelix Genome Browse software (available at: http://goldenhelix.com/GenomeBrowse/) and the Ensemble Genes 61 reference set. Prediction of regulatory elements was performed by lifting over CanFam2.0 coordinates to the Human GRCh37/hg19 assembly by orthology, and annotating ENCODE tracks in the UCSC genome browser (available at: http://genome.ucsc.edu/).

## Supporting Information

S1 File Supporting phenotype and genotype data. (ZIP)
